# Have Birds the Faculty of Articulate Speech?

**Published:** 1881-10

**Authors:** William A. Hammond


THE JOURNAL

OF

COMPARATIVE MEDICINE AND SURGERY.

VOL. II. OCTOBER, 1881. NO. 4.

### ORIGINAL COMMUNICATIONS.

### ART. XV.—HAVE BIRDS THE FACULTY OF ARTICULATE SPEECH?

THAT birds, like other animals, have the power of conveying ideas by sounds scarcely admits of a doubt, and that others are capable of learning words from human languages, is well known by the familiar examples afforded by ravens, parrots, etc. The question which I shall ask, however, is: Do they have the faculty of communicating ideas to each other by means of words analogous to the faculty of speech possessed by man? It may be impossible, at present, to answer this question; but there are facts in our possession which go far towards its elucidation, and some of the more important of these I propose to bring to the notice of the readers of this journal.

Bechstein* has very thoroughly studied the notes of the nightingale, and has ascertained that this bird, in the course of an hour or two, enunciates over forty distinct sounds, which are representable by letters of our alphabet. In the vocabulary of this sweetest of all singing birds there are double consonants, vowels and double vowels.

---

* Naturgeschichte der Hof und Stuben vogel Art. Nachtigall.

In attempting to represent the song of the nightingle, Bechstein writes it as follows, the letters having the sounds given to them in the German language: "Tiore, tiore, tiore, tiore—Spe, tiore, aqua—Tio, tio, tio, tio, tio, tio, tio, tio, tix—Contio, contio, contio, contio—Squo, squo, squo, squo—Tzu, tzu, tzu, tzu, tzu, tzu, tzu, tzu, tzu, tzi—Corror, tiou, squa, pipi—qui—Zozozozozozozozozozozozo, zirrkading!—Tsissiri, Tsipisisisisisis—Dzorre, dzorre, dzorre, dzorre, hi—Tzatu, tzatu, tzatu, tzatu, tzatu, tzatu, tzatu, dzi—Dlo, dlo, dlo, dlo, dlo, dlo, dlo, dlo, dlo—Quio trrrrrrrr itz—Lu, lu, lu, lu, ly, ly, ly, ly, lie, lie, lie, lie—Quio, didl, li, lubylie—Hagur, gurr, quihio, coui, coui, coui, coui, qui, qui, qui, qui, qui, qui, qui—Goll, goll, goll, goll, guia hadadoi—Couigui, horr, ho, didia, dill, ei—Hezezezezezezezezezezezezezezeze couar ho dze hor—Quia, quia, quia, quia, quia, quia, quia, quia, ti—Ki, ki, ki, io, io, io, ioioioio, ki—Lu ly li le lai la leu lo didl io quia—Kigaigaigaigaigaigaigaigai, guiagaigaigai coruor dzio dzio pi.

Here we find all the vowels of the languages of European nations, and all the consonants except *b*, *f*, *m*, *n* and *v*. It would be presumption for us to deny that the syllables enunciated are entirely without meaning, and that the nightingles do not understand the sounds that are uttered by their fellows. The idea that the song of the nightingle is nothing but a song, without any other motive than the gratification of the musical taste of the singer, appears to me to be entirely unsupported by analogy, either when compared with man or with other animals. All sounds, musical or not, though occasionally emitted for self-gratification, have no such object as their primary motive. This latter is the influencing of others, either for the purpose of alarming, pleasing, or moving them in some other way; and it would be, I think, unwarrantable to deny the existence of a like consideration in birds, and this especially when so elaborate a system as that of the nightingle is in existence.

In his remarks on the subject of the vocalization of which birds are capable, Houzeau says:

"The language of birds contains a notable part of the letters of the alphabet, and even the double consonants are employed, such as *dz* and *db*. There are also simple vowels and diphthongs. The greater part of the consonants used are combined with vowels as initial letters of syllables. The use of consonants after vowels, as final letters, is much more rare, the letters *l*, *n* and *r* being the only ones which figure in this situation. But, from the lack of various letters and of combinations or syllables, we are not to conclude that there is a total absence of articulate sounds. We ourselves do not make use of all possible combinations of final consonants after a vowel. Many nations do not even use all the letters, and cannot even pronounce those which do not enter into the composition of their alphabets. Several African languages do not contain *r*. The Chinese not only do not have this rolling consonant, but they lack also other simple consonants, as *b*, *d*, *v*, *z*. They pronounce Holland *Golanki*, and France *Fulantsus*. The Polynesians do not have the sibilant *s*. They do not place consonants after vowels in the same syllables, so that all their words end in vowels. The Hurons have none of the labials (*b*, *f*, *m*, *n*, *p*, *v*), and the vowel *u*, which is articulated by the lips, is also wanting. Garcilasso says that the Peruvians not only do not have the letters *b*, *d*, *f*, *g*, *z*, *x*, but that they do not even form a single double consonant. The Feejeans do not possess the *c*; the Pomo Somos the *k*, or the inhabitants of Rakiraki the *t*. The Australians have no *s*. The Indians of *Port au Francais*, in British Columbia, lack the letters *b*, *d*, *f*, *j*, *h*, *v*, *x*. But the poorest of all people in articulate sounds are those of New Zealand, whose alphabet does not contain the twelve following letters: *b*, *c*, *d*, *f*, *g*, *j*, *l*, *q*, *s*, *v*, *y*, *z*, and the double letter *x*."

Thus we see that the nightingle possesses a larger literary capital than do several nations of men, and I therefore submit that there is nothing at all improbable in the view that it has also the faculty of communicating, by means of articulate speech, the ideas which its brain elaborates. It must be borne in mind that the cerebral structure of birds is simple when compared to that of higher animals. In proportion to the size of its body, however, the brain of the nightingale is large; and that part which is in man supposed to be connected with the faculty of speech, is more developed than the corresponding part in other birds.

That birds, in common with other animals, convey ideas by sounds no one probably disputes ; but I contend that in all probability they go beyond this, and approach, though of course remotely, the powers of man in this respect.

I venture to conclude with the following anecdote, which I quote from Houzeau, who accepts it as true :

A farmer on Long Island, in the State of New York, wounded a wild goose as it was passing south on its winter migration. The bird was soon cured of its injury, and was content to remain with the tame geese belonging to the farmer. In the spring, one day, as a large flock of wild geese were flying over the farm on their journey north, the bird heard their cries, and at once took flight and joined them. But the following autumn the wild geese suddenly appeared among the tame ones of the farm. One was the wounded goose, which had returned, bringing two others with it, and they all remained with the companions of their choice.

WILLIAM A. HAMMOND, M.D.

